# Contextual Effect of Wealth on Independence: An Examination through Regional Differences in China

**DOI:** 10.3389/fpsyg.2016.00384

**Published:** 2016-03-17

**Authors:** Kosuke Takemura, Takeshi Hamamura, Yanjun Guan, Satoko Suzuki

**Affiliations:** ^1^Faculty of Economics, Shiga UniversityHikone, Japan; ^2^School of Psychology and Speech Pathology, Curtin UniversityPerth, WA, Australia; ^3^Business School, Durham UniversityDurham, UK; ^4^Graduate School of Management, Kyoto UniversityKyoto, Japan

**Keywords:** culture, independence, individualism, economic development, multilevel, contextual effect

## Abstract

The current study disentangled two different effects of wealth on psychological tendency toward independence: one is an effect exerted at the individual level (i.e., being rich) and the other one is a contextual effect (i.e., being surrounded by rich individuals). Past research has found a stronger tendency toward independence among people in economically developed societies. This association has often been explained as a result of a greater amount of choices, and thus more opportunities to express individuality that wealth affords individuals. In addition to this individual-level process, theories in cultural psychology imply that the wealth-independence link also reflects social processes—living in a rich society, regardless of one’s own wealth, promotes independence (contextual effect of wealth on independence). Through a large-scale survey in China, using multilevel analyses, we found that wealth had both the individual-level effect and contextual effect on independence as well as related psychological tendencies (influence orientation and generalized trust), suggesting that individuals are more likely to be independent with greater personal wealth and when surrounded by wealthy others. Possible processes through which independence is promoted by liing in a wealthy area are discussed.

## Introduction

Across human societies, individuals differ in their relative independence from, and interdependence within, social relationships (Hofstede, 1980; Markus and Kitayama, 1991; Triandis, 1995; see also Henrich et al., 2010b). Independent (individualistic) tendency is characterized by individuals seeing themselves as independent from others, which also relates to motivation to influence (rather than adjusting oneself to) others (Morling et al., 2002) and willingness to interact with others outside of one’s immediate social circles, and thus generalized trust of others (Allik and Realo, 2004; Hamamura, 2012). These psychological tendencies give rise to behavioral patterns that, in turn, collectively maintain the social environment and reinforce psychological processes associated with independence (Markus and Kitayama, 1991; Shweder, 1991; Leung and Cohen, 2011; Yamagishi, 2011).

One of the best-established correlates of independent tendency at the societal level is wealth. Previous cross-cultural research has found that people residing in rich societies are more likely to show independent tendencies (Hofstede, 1980; Inglehart and Baker, 2000; Greenfield et al., 2003; Georgas et al., 2004; Grossmann and Varnum, 2015). For example, Hofstede (1980) reported a strong correlation (*r* = 0.82) between Gross National Product per capita and individualism through a nation-level correlation analysis. Georgas et al. (2004) found that differences in individualism across countries was best explained by economic differences among a range of eco-cultural variables, such as religion and education.

However, the societal-level correlation between wealth and independence found in previous studies (e.g., Hofstede, 1980) may either reflect an effect of aﬄuent societies promoting independent tendencies or an effect of aﬄuent individuals tending to be more independent, or both of them. Stated differently, the individual-level effect of wealth may give rise to the societal-level pattern that Hofstede (1980) identified.

In fact, a number of theories have interpreted the wealth-independence link as reflecting the effects associated with personal wealth. For example, it has been suggested that wealth promotes independence because “(a)ﬄuence implies the ability to ‘do one’s own thing”’ (Triandis et al., 1988, p. 324) or “aﬄuence creates choices and allows people to express their individuality by making different choices” (Schimmack et al., 2005, p. 21). This type of explanation postulates that personal wealth facilitates greater independence through its effect in affording more choices to individuals. Consistent with this theory, research has demonstrated an association between individual differences in socioeconomic status and independence (e.g., Kohn et al., 1990; Stephens et al., 2007; Kraus and Keltner, 2009; Grossmann and Varnum, 2010; Na et al., 2010; Hamamura et al., 2013).

On the other hand, influential theories in cultural psychology seem to support the view that wealth also has a unique effect on independence at the societal level. Theories of cultural psychology have emphasized roles of social processes, such as shared meaning systems (Markus and Kitayama, 1991; Snibbe and Markus, 2005; Kitayama and Uskul, 2011), social norms (Gelfand et al., 2011), and shared social contexts (Kraus et al., 2012) in shaping psychological processes. These social processes may help explain the effect of wealth on independence beyond its effect operating at the level of individuals. In other words, these theories predict the presence of a contextual effect, that wealth exerts an effect on independence at the societal level above and beyond its effect at the individual level. Depending on the relative strength of individual-level and societal-level effect, the contextual effect can take two forms (e.g., Tay et al., 2014). One is the case in which the societal-level effect is larger than the individual-level effect—a society’s wealth has a stronger effect on people’s independence than predicted on the basis of their level of aﬄuence. The other is the case in which the societal-level effect is smaller than the individual-level effect—a society’s wealth has a weaker effect on people’s independence than predicted on the basis of their aﬄuence. Theories in cultural psychology seem to suggest the presence of the former type of contextual effect. Analytically, a contextual effect can be computed in a multilevel model that simultaneously examines the effect of wealth on independence at the level of individual (within-group effect) and society (between-group effect) and by taking their difference (Raudenbush and Bryk, 2002).

In some areas of research, contextual effects have clearly been established (e.g., Henrich et al., 2005, 2010a; Christ et al., 2014; Colleran et al., 2014; Tay et al., 2014). For example, findings in Henrich et al. (2005, 2010a) suggested that anonymous interpersonal interactions that are normative in modern market exchange tend to promote community members’ prosocial behavior toward an anonymous other, above and beyond the effects associated with individuals’ personal experiences of market exchange. This research demonstrated the role of a socially shared factor (the norms of anonymous interactions at markets) in influencing individual behavior (prosocial behavior toward an anonymous other) beyond the individual-level process (personal experience of market exchange).

In the current study, we predicted a significant individual-level association between wealth and independence, replicating past research (e.g., Kohn et al., 1990; Stephens et al., 2007; Kraus and Keltner, 2009; Grossmann and Varnum, 2010; Na et al., 2010; Hamamura et al., 2013). Additionally, we examined wealth’s contextual effect on independent tendency—an effect suggested by cultural psychological theories articulating the role of social processes in shaping psychological processes (Markus and Kitayama, 1991; Gelfand et al., 2011; Kitayama and Uskul, 2011).

We examined the effect of wealth on independent tendency across provinces in China. China was an excellent context for the current research because there are large regional variations within China with regard to the distribution of wealth (Yang, 2002; Fleisher et al., 2010), as well as in the cultural practices maintained through legal restrictions on inter-provincial migrations. Yet, because the provinces are parts of a single nation, major variables that usually confound cross-country comparisons are naturally ruled out (Varnum et al., 2010).

The units of analysis in the current study were individual participants (Level 1) that are nested within provinces (Level 2). Participants were recruited from all the provinces in mainland China, who provided information on their annual household income and responded to scales designed to measure their tendency toward independence. One measure of independence was the Independent Self-Construal Scale (Singelis, 1994). In addition to this direct measure of independence, we assessed two psychological tendencies conceptually associated with independence. One measure assessed orientation toward influencing surrounding social circumstances (as opposed to adjusting oneself). As the independent self is perceived as relatively immutable and a consistent entity working within the context of a mutable world (Heine, 2008), individuals with independent self-construal tend to engage in influence rather than adjustment (Morling et al., 2002). The other measure assessed generalized trust or trust toward others who are not closely connected to oneself (Yamagishi and Yamagishi, 1994). Previous studies (Allik and Realo, 2004; Hamamura, 2012) found a positive association between independence and generalized trust, suggesting that taking the risk of trusting others outside of a close-knit community may be sustained by viewing oneself as independent from others [but see also the findings of Yuki et al. (2005) showing that individualists sometimes show a strong in-group bias; see Brewer and Chen (2007), and Brewer and Yuki (2007), for reviews]. Thus, we explored if generalized trust would show similar correlational patterns as independent self-construal.

We performed multilevel analyses using group-mean centering. Hence, all the predictors at the individual level were centered around respective provincial means. This method removes all inter-provincial differences and allows examination of within-province (individual-level) effects and between-province (province-level) effects separately from each other (Enders and Tofighi, 2007). A contextual effect is obtained by taking the difference between these two effects. The positive contextual effect (between-province > within-province effect) would indicate that wealth at the level of provinces has a stronger effect on people’s independence than predicted on the basis of people’s aﬄuence.

## Materials and Methods

### Participants

Participants were recruited from all the provinces (including directly controlled municipalities as well as autonomous regions) in mainland China by a marketing research firm, Cross Marketing, Inc. Fifty individuals from each province took part in the study, except for Shanghai and Gansu from which 100 people participated. (The larger sample size from these locations is for the purpose of another research project in which the current study was part.) The total number of participants was 1,650, which is comparable to a previous study examining regional differences in China (Talhelm et al., 2014, *N* = 1,162). In all provinces, the same recruiting methods were used (i.e., advertising on the Internet, mass media, and face-to-face surveys). The sample size at both levels were determined prior to data collection. To maximize the sample size at level 2 (province level), which is essential for accurate estimations of the level 2 effects (Maas and Hox, 2005), we collected data from all the provinces in mainland China (i.e., *n* = 31). The number of participants from each province (*n* = 50 for most provinces) was determined based on results of simulations by Maas and Hox (2005) to ensure unbiased estimations in multilevel analyses. Participants’ responses were collected through an online survey (Supplementary Table S1 for sample characteristics).

### Individual-Level Measures

Participants completed scales designed to measure independence and related tendencies. The scales were the Self-Construal Scale (Singelis, 1994), Circumplex Scales of Interpersonal Values (CSIV; Locke, 2000), and Generalized Trust Scale (Yamagishi and Yamagishi, 1994). All scales were administered in Chinese. The consensus method was taken to ensure the validity of translations, and at least two independent translators checked the translations (see Supplementary Table S1 for scale reliability and intra-class correlations of the measures).

The Self-Construal Scale consisted of 12 items for assessing independent self-construal (e.g., “*Being able to take care of myself is a primary concern for me*”) and 12 items for assessing interdependent self-construal (e.g., “*I will sacrifice my self-interest for the benefit of my group*”). Responses were indicated on a 7-point scale with options ranging from 1 (*strongly disagree*) to 7 (*strongly agree*).

The CSIV assessed influence–adjustment orientation. Participants were instructed to think about interpersonal situations (such as being with close friends, with strangers, at work, and at social gatherings) and indicate, for example, how important it was to them “*that I have an impact on them*” (influence) and “*that I go along with what they want to do*” (adjustment). Following the procedure used in previous studies (Tsai et al., 2007; Miyamoto and Wilken, 2010), four items were used to measure influence orientation and five items were used to measure adjustment orientation. Responses were indicated on a 5-point scale with options ranging from 0 (*not important to me*) to 4 (*extremely important to me*).

The third measure, the Generalized Trust Scale, included five items such as, “*Most people are basically honest*.” Responses were indicated on a 7-point scale with options ranging from 1 (*strongly disagree*) to 7 (*strongly agree*).

After completing these psychological measures, participants provided information on their annual household income (open-ended format), which was log-transformed prior to analyses in order to correct the skew. They also indicated their gender, age, and education level. For education level, participants were asked to indicate their latest academic background from a list of options ranging from elementary school to graduate school (Ph.D. level). We used a dummy-coded variable for education (reference category = no college degree) in our analyses.

The institutional review board at Kyoto University [the primary investigator’s (KT) home institution at the time of study] did not require review of a low-risk survey such as ours. As such, we did not seek institutional approval. All aspects of the study were conducted in accordance with the ethical rules of the American Psychological Association. Participation was voluntary and consent was indicated by completion of the survey.

### Province-Level Measures

As an index of province-level wealth, we used provincial means of participants’ annual household income (after log-transformation). We also obtained several province-level variables and included them as covariates. These included population density (Vandello and Cohen, 1999) and inequality in the distribution of wealth (Fincher et al., 2008). We computed population density from population size (obtained from National Bureau of Statistics of China, 2011) divided by land area (obtained from the website of the State Council of the People’s Republic of China^1^ on February 5, 2014). For an index of inequality, urban-rural income disparity was used (i.e., ratio of urban disposable income to rural net income, obtained from All China Data Center^2^ on February 6, 2014). In addition, the amount of direct foreign investments into provinces (obtained from All China Data Center on February 6, 2014) was controlled for, because greater foreign investment may signal greater exposure to Western cultures (Yang, 2002) and may contribute to independent tendencies of residents. Percentage of in-migrants from other provinces (obtained from Duan et al., 2013) was also included in the models, as past research suggested that high residential mobility promotes independent tendencies (Oishi and Kisling, 2009) and wealthy provinces presumably attract more migrants. Additionally, because four directly controlled municipalities in China (i.e., Beijing, Chongqing, Tianjin, and Shanghai) are generally urbanized, we included a dummy-coded variable to represent a directly controlled municipality. Finally, as an index of education at the province level, we calculated the percentage of those who obtained a college degree or above from our participants’ responses. We included this variable as a covariate at the province level. Descriptive statistics of the province-level variables are shown in Supplementary Table S2.

## Results

### Zero-Order Correlations

As preliminary analyses, we examined zero-order correlations at the individual level (**Table 1**) and at the province level (**Table 2**). As anticipated, log-transformed annual income (individual level) was positively associated with independent self-construal (*r* = 0.22), influence orientation (*r* = 0.26), and generalized trust (*r* = 0.12).

**Table 1:** Zero-order correlations between individual-level variables (Pearson’s *r*).

	(2)	(3)	(4)	(5)	(6)	(7)	(8)	(9)
(1) Annual income (log-transformed)	-0.01	0.06^∗∗^	0.24^∗∗∗^	0.22^∗∗∗^	0.12^∗∗∗^	0.26^∗∗∗^	0.16^∗∗∗^	0.12^∗∗∗^
(2) Gender (ref = male)		-0.16^∗∗∗^	0.01	-0.08^∗∗^	-0.06^∗^	-0.08^∗∗∗^	-0.11^∗∗∗^	-0.01
(3) Age			-0.15^∗∗∗^	0.18^∗∗∗^	0.10^∗∗∗^	0.00	-0.03	0.13^∗∗∗^
(4) Education (ref = no college degree)				0.05^∗^	0.08^∗∗∗^	0.18^∗∗∗^	0.13^∗∗∗^	0.06^∗^
(5) Independent self-construal					0.66^∗∗∗^	0.54^∗∗∗^	0.41^∗∗∗^	0.42^∗∗∗^
(6) Interdependent self-construal						0.44^∗∗∗^	0.42^∗∗∗^	0.50^∗∗∗^
(7) Influence							0.63^∗∗∗^	0.31^∗∗∗^
(8) Adjustment								0.24^∗∗∗^
(9) Generalized trust								

*^∗∗∗^p < 0.001, ^∗∗^p < 0.01, ^∗^p < 0.05.*

**Table 2:** Zero-order correlations at the province level (Pearson’s *r*).

	(2)	(3)	(4)	(5)	(6)	(7)	(8)	(9)	(10)	(11)	(12)
(1) Provincial mean of logged annual household income	0.45^∗^	0.64^∗∗∗^	0.66^∗∗∗^	-0.50^∗∗^	0.53^∗∗^	0.39^∗^	0.60^∗∗∗^	0.25	0.62^∗∗∗^	0.49^∗∗^	0.41^∗^
(2) Directly controlled municipality		0.67^∗∗∗^	0.43^∗^	-0.34^†^	0.16	0.44^∗^	0.22	0.10	0.20	0.15	-0.09
(3) Population density (person/km^2^)			0.64^∗∗∗^	-0.47^∗∗^	0.38^∗^	0.38^∗^	0.37^∗^	0.17	0.33^†^	0.24	0.14
(4) Percentage of in-migrants				-0.40^∗^	0.37^∗^	0.41^∗^	0.39^∗^	0.18	0.29	0.18	0.21
(5) Ratio of rural net income to urban disposable income					-0.49^∗∗^	-0.34^†^	-0.23	-0.01	-0.18	-0.04	-0.40^∗^
(6) Direct foreign investments (1 million USD)						0.23	0.17	0.10	0.20	0.16	0.23
(7) Percentage of person with college degree or above							0.29	0.39^∗^	0.50^∗∗^	0.46^∗∗^	0.41^∗^
8) Independent self-construal								0.71^∗∗∗^	0.81^∗∗∗^	0.72^∗∗∗^	0.59^∗∗∗^
(9) Interdependent self-construal									0.72^∗∗∗^	0.76^∗∗∗^	0.57^∗∗∗^
(10) Influence										0.90^∗∗∗^	0.67^∗∗∗^
(11) Adjustment											0.62^∗∗∗^
(12) Generalized trust											

*^∗∗∗^p < 0.001, ^∗∗^p < 0.01, ^∗^p < 0.05, ^†^p < 0.10.*

Unexpectedly, interdependent self-construal (*r* = 0.12) and adjustment orientation (*r* = 0.16) were also positively correlated with annual income, but to a somewhat lesser degree when compared to the counterpart subscale (i.e., independent self-construal for interdependent self-construal, and influence orientation for adjustment orientation). The unexpected positive correlations between annual income and the interdependence measures might be caused by response style, such as acquiescence response style, which has been found to be strong in collectivistic societies (Schimmack et al., 2005). In fact, the counterpart subscales were positively correlated (*r* = 0.66 for independent and interdependent self-construal, *r* = 0.63 for influence and adjustment orientations), though past research suggests that independence and interdependence are two separate constructs (Oyserman et al., 2002). As such, in our main analyses using multilevel modeling the counterpart subscale was controlled for. For example, independent self-construal was analyzed using the residual term of the regression analysis in which independent self-construal was predicted from interdependent self-construal. Similarly, when we predicted influence we controlled for adjustment by using the residual from the regression of influence on adjustment. After controlling for the counterpart subscales, correlational patterns between the scales were in line with theoretical predictions. Independent self-construal was positively correlated with influence, *r* = 0.28, *p* < 0.001, while it was very weakly (and negatively) correlated with adjustment, *r* = –0.04, *p* = 0.069. Also, interdependent self-construal was not correlated with influence, *r* = –0.02, *p* = 0.356, while it was positively correlated with adjustment, *r* = 0.17, *p* < 0.001.

At the province level (**Table 2**), provincial mean of logged annual household income was positively associated with independent self-construal (*r* = 0.60), influence (*r* = 0.62), and generalized trust (*r* = 0.41). Unexpectedly, income was also positively associated with adjustment, although relatively weakly (*r* = 0.49). Provincial income correlated with several province-level variables (e.g., population density, percentage of in-migrants). As these correlations among the province-level variables might be confounding the correlations between provincial income and the independence-related measures, in the following analyses we controlled for the effects of these province-level factors as well as the individual-level factors (i.e., age, gender, and education level). Controlling for these variables was important in order to examine the unique effect of wealth on the independent tendencies at the individual level as well as the province level using multilevel modeling. Among the province-level covariates, directly controlled municipality, population density, and percentage of in-migrants—three variables presumably related to degree of urbanization—were highly correlated with each other (*r*s = 0.43 to 0.67). To avoid multicollinearity in the multilevel modeling, we created a single composite measure of urbanity by averaging them after standardization, and used the composite measure as a province-level covariate.

### Multilevel Analyses of Independence

Multilevel analyses were conducted predicting independence and controlling for the individual- and province-level covariates (**Table 3**). We found that the within-province effect of annual income on independence was significant and positive. This finding confirms the wealth-independence association at the individual level. Similarly, within-province effects of annual income were also found for influence orientation and generalized trust. The between-province effects of income were also found to be significant and positive on all the independence-related measures.

**Table 3:** Multilevel analyses of independent self-construal, influence, and generalized trust.

	Independent self-construal^1^			Influence^2^			Generalized trust		
	Coefficient	*SE*	*p*	Coefficient	*SE*	*p*	Coefficient	*SE*	*p*
Annual income (log-transformed)								
Within-province effect	**0.28**	(0.04)	<0.001	**0.31**	(0.05)	<0.001	**0.26**	(0.08)	0.001
Between-province effect	**0.93**	(0.22)	<0.001	**1.18**	(0.26)	<0.001	**1.00**	(0.38)	0.014
Contextual effect	**0.65**	(0.22)	0.008	**0.87**	(0.27)	0.003	**0.74**	(0.39)	0.066
Individual-level covariates									
Gender (ref = male)	-0.04	(0.03)	0.197	-0.03	(0.03)	0.381	0.03	(0.05)	0.550
Age	**0.01**	(0.00)	<0.001	0.00	(0.00)	0.537	**0.01**	(0.00)	<0.001
Education (ref = no college degree)	-0.03	(0.03)	0.238	**0.11**	(0.03)	0.001	**0.09**	(0.05)	0.076
Province-level covariates								
Ratio of rural net income to urban disposable income	-0.04	(0.03)	0.245	0.00	(0.04)	0.930	**-0.11**	(0.06)	0.065
Direct foreign investments (10,000 USD)	**-0.0000001**	(0.00)	0.066	0.00	(0.00)	0.215	0.00	(0.00)	0.547
Urbanity^3^	0.00	(0.03)	0.968	**-0.06**	(0.03)	0.087	**-0.12**	(0.04)	0.009
Percentage of person with college degree or above	**-0.33**	(0.18)	0.087	**0.53**	(0.22)	0.024	**0.74**	(0.32)	0.028
Model fit and summary								
Sample size	1644			1644			1644		
AIC	2580			3071			4501		
BIC	2644			3135			4566		
Log likelihood	-1278			-1523			-2238		

*Estimation method: maximum likelihood. All individual-level predictors centered around province-mean. Effects with p < 0.10 appear in bold print. ^1^The residual was used from the regression of independent self-construal on interdependent self-construal. ^2^The residual was used from the regression of influence on adjustment. ^3^Urbanity is a composite measure of Directly controlled municipality (dummy-coded), Population density, and Percentage of in-migrants (averaged after standardization).*

**FIGURE 1:**
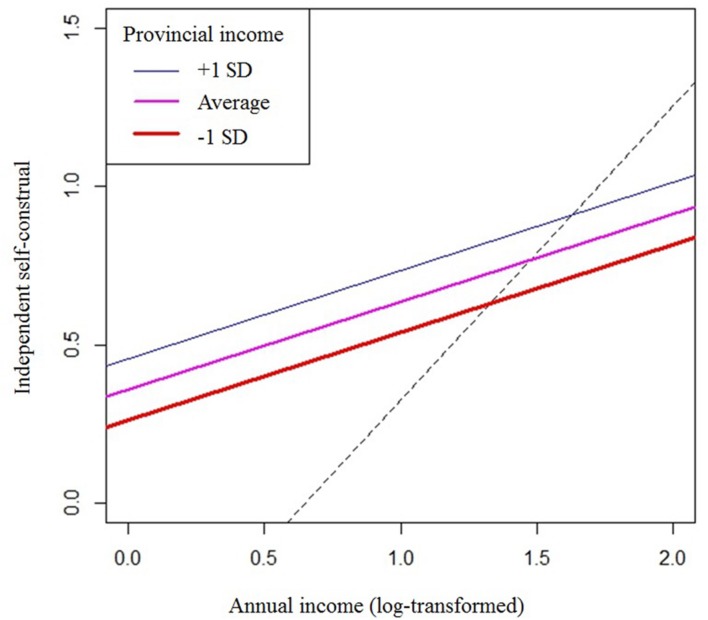
**Results of the multilevel analysis: Independent self-construal as a function of logged annual household income.** In the graph, the three solid lines are regression lines for the effect of individual-level income on independent self-construal at three levels of provincial income (average household income at the province level). The dotted line is the regression line for the relationship between province-level income and independent self-construal.

Importantly, the contextual effect, which was obtained by taking the difference of the between-province and within-province effect, was positive in all cases. As illustrated in **Figure 1**, the regression line for the between-province effect of income on independent self-construal was steeper than the regression lines for the within-province effect of income, and this contextual effect (i.e., the difference between within-province and between-province effects) was positive and significantly different from zero. Similar patterns were found for influence and generalized trust, though the contextual effect on generalized trust was relatively weak and marginally significant (**Table 3**).

In summary, we found consistent patterns across all the independence-related tendencies. Annual income had positive associations with independence and related tendencies above and beyond the individual level. Among participants with the same level of personal wealth, those in rich provinces were more likely to be independent, influence-oriented, and trustful of others, than those in less rich provinces.

### Supplementary Analysis: Effect of Wealth on Interdependence

We also examined the effects of wealth on the interdependence-related tendencies by similar multilevel analyses (**Table 4**). As expected, after controlling for various possible confounding factors the associations between wealth and the interdependent measures, which were partially observed in the zero-order correlation analyses, were no longer found. Annual income was not associated with interdependent self-construal or adjustment orientation either at the individual or province level. The diverging patterns of findings between independence and interdependence-related tendencies are consistent with an observation in the literature that independent and interdependent tendencies are separate dimensions with different correlates (Oyserman et al., 2002).

**Table 4:** Multilevel analyses of interdependent self-construal and adjustment.

		Interdependent self-construal^1^				Adjustment^2^		
		Coefficient	*SE*	*p*		Coefficient	*SE*	*p*
Annual income (log-transformed)								
	Within-province effect	-0.06	(0.04)	0.161		0.00	(0.05)	0.946
	Between-province effect	-0.40	(0.24)	0.115		-0.04	(0.23)	0.867
	Contextual effect	-0.34	(0.25)	0.187		-0.04	(0.23)	0.880
Individual-level covariates								
	Gender (ref = male)	-0.01	(0.03)	0.777		**-0.09**	(0.03)	0.002
	Age	0.00	(0.00)	0.785		0.00	(0.00)	0.115
	Education (ref = no college degree)	**0.08**	(0.03)	0.009		0.01	(0.03)	0.719
Province-level covariates								
	Ratio of rural net income to urban disposable income	0.06	(0.04)	0.121		0.04	(0.03)	0.196
	Direct foreign investments (10,000 USD)	0.00	(0.00)	0.220		0.00	(0.00)	0.567
	Urbanity^3^	-0.01	(0.03)	0.734		-0.01	(0.03)	0.812
	Percentage of person with college degree or above	**0.59**	(0.21)	0.008		0.15	(0.19)	0.428
Model fit and summary								
	Sample size	1644				1644		
	AIC	2586				2846		
	BIC	2651				2911		
	Log likelihood	-1281				-1411		

*Estimation method: maximum likelihood. All individual-level predictors centered around province-mean. Effects with p < 0.10 appear in bold print. ^1^The residual was used from the regression of interdependent self-construal on independent self-construal. ^2^The residual was used from the regression of adjustment on influence. ^3^Urbanity is a composite measure of Directly controlled municipality (dummy-coded), Population density, and Percentage of in-migrants (averaged after standardization).*

## Discussion

Previous studies have found that individuals living in rich societies are inclined toward independence. The purpose of the current study was to separate the individual-level effect and contextual effect of wealth on independence, and to examine whether the wealth-independence link emerges through social processes above and beyond individual processes. We investigated regional differences in independence-related tendencies (independent self-construal, influence orientation, and generalized trust) across 31 provinces in China. The multilevel analyses confirmed the individual-level associations between wealth and independence-related tendencies. The results also revealed that income had a contextual effect on independence-related tendencies, as indicated by larger effects at the province level than the individual level. Thus, even if one is not rich, his or her independent tendency is likely promoted through the aﬄuence of surrounding others.

Below, we discuss a question that should be addressed in future studies. Specifically, why does wealth of surrounding others promote one’s independence? The first possibility concerns the social transmission of psychological/behavioral tendencies from rich to non-rich individuals. It has been suggested that people tend to learn from prestigious others (e.g., Colleran et al., 2014). Rich (i.e., economically successful) individuals may be regarded as a model of social learning. Consequently, individuals living in rich provinces might have acquired independent tendency through social learning from rich people, who have acquired independent tendency based on their own richness through individual-level processes.

The second possibility is based on the social norms and institutions, particularly norms of modern market exchange, that people create as a group. These norms may promote independent tendency by facilitating interactions among strangers (Greenfield et al., 2003; Henrich et al., 2010a). At the individual level, the extent to which an individual is integrated into market exchange (e.g., reliant on markets in obtaining resources) may be correlated with the amount of money he or she has, as money affords means to obtain resources through market exchange. Consider a case of labor outsourcing, such as baby-sitting. One could either arrange childcare through their own social network (e.g., asking a friend or a relative) or through market exchange, and rich individuals are more able to rely on the latter. At the societal level, presence of a large number of market-integrated individuals likely indicates the salience of social norms relevant to exchange with strangers, which in turn may promote independent tendency (Yuki and Takemura, 2013). Research supports this rationale. For example, members of herding communities, in comparison to farming communities, interact with strangers more (Uskul and Over, 2014) and have more independent tendency (Witkin and Berry, 1975). Similarly, Leung and Cohen (2011) argued that the presence of modern markets is connected to a culturally shared notion that individual worth is inalienable (vs. socially conferred), which is an important aspect of independent culture (Heine, 2005). These findings imply that in provinces where many individuals are engaged in market exchange and use money to obtain resources, norms that facilitate independent tendency are collectively created.

The third explanation is the role of choice that wealth affords at the individual level (e.g., Triandis et al., 1988). While originally this theory might have suggested that it is the amount of choices that an individual *has* that matters, our findings suggest that what matters more is the amount of choices that an individual *sees*. Presumably, there are more shops, goods, and services in the wealthier provinces, and individuals would perceive a greater range of options and possibilities for goods and services to select from in richer provinces regardless of one’s own richness. In turn, perceiving a wider range of options and possibilities may motivate individuals to pursue personal success and achievement over interpersonal harmony with others.

In summary, the current study contributes to the literature by identifying the role of social processes in the wealth-independence link. The current findings, in turn, shed light on a new question: What are the specific social mechanisms underlying the wealth-independence link? Further research on this issue would be instrumental in advancing understanding of the association between wealth and independent psychological processes.

## Author Contributions

All authors contributed to the study design. Data collection and the data analysis were performed by KT. KT and TH drafted the manuscript, and YG and SS provided critical revisions. All authors approved the final version of the manuscript for submission.

## Conflict of Interest Statement

The authors declare that the research was conducted in the absence of any commercial or financial relationships that could be construed as a potential conflict of interest.

## Footnotes

1 http://english.gov.cn/

2 http://chinadataonline.org
