# Supplementary material for: Contextual Effect of Wealth on Independence: An Examination through Regional Differences in China
Source: Front Psychol. 2016 Mar 17;7:384. doi: 10.3389/fpsyg.2016.00384 (PMC4794504; doi:10.3389/fpsyg.2016.00384)
Supplement: Supplementary file 1 [file Data_Sheet_1.DOCX]

Supplementary Material

Contextual effect of wealth on independence: an examination through regional differences in China

Kosuke Takemura*, Takeshi Hamamura, Yanjun Guan, Satoko Suzuki

*** Correspondence:** Kosuke Takemura: boz.takemura@gmail.com

**Table S1. Sample characteristics**

|  |  | **M** | **Med** | **(SD)** | **Min** | **Max** | **Prop** | **α** | **ICC** |  |
| --- | --- | --- | --- | --- | --- | --- | --- | --- | --- | --- |
| ***Individual level*** | |  |  |  |  |  |  |  |  |  |
|  | Independent self-construal | 4.86 | 4.83 | (0.73) | 1.42 | 7.00 |  | .81 | .03 | *** |
|  | Interdependent self-construal | 4.97 | 5.00 | (0.71) | 1.33 | 7.00 |  | .82 | .02 | *** |
|  | Influence | 2.11 | 2.00 | (0.81) | 0.00 | 4.00 |  | .77 | .06 | *** |
|  | Adjustment | 1.89 | 1.80 | (0.74) | 0.00 | 4.00 |  | .69 | .02 | *** |
|  | Generalized trust | 5.01 | 5.00 | (0.96) | 1.00 | 7.00 |  | .83 | .01 | ** |
|  | Annual household income (10 thousand CNY) ^1)^ | 28.00 | 10.00 | (497.72) | 0.00 | 20,000.00 |  |  | .09 | *** |
|  | Female |  |  |  |  |  | 0.41 |  |  |  |
|  | Age | 32.86 | 31.00 | (8.67) | 16.00 | 66.00 |  |  |  |  |
|  | College degree or above |  |  |  |  |  | 0.64 |  |  |  |
| ***Province level*** ^2)^ | |  |  |  |  |  |  |  |  |  |
|  | Directly controlled municipality |  |  |  |  |  | 0.12 |  |  |  |
|  | Population density (person/km^2^) | 430.99 | 267.03 | (661.43) | 2.50 | 3,630.79 |  |  |  |  |
|  | Ratio of rural net income to urban disposable income | 3.02 | 2.95 | (0.54) | 2.19 | 4.07 |  |  |  |  |
|  | Direct foreign investments (1 million USD) | 5,816.53 | 4,050.15 | (6,782.34) | 24.34 | 28,497.77 |  |  |  |  |
|  | Percentage of in-migrants | 15.24 | 10.43 | (8.92) | 7.29 | 40.99 |  |  |  |  |
| **p* < .05, ***p* < .01, ****p* < .001 | |  |  |  |  |  |  |  |  |  |
| *Note*. Intra-class correlations were calculated by HAD11 (Shimizu, Murayama, & Daibo, 2006).  ^1)^ Log-transformed annual income was used to calculate ICC. ^2)^ All province-level data were from 2010. | | | | | | | | | | |

**Table S2. Sample characteristics of the 31 provinces**

| Province | Number of participants | Annual household income (Mean/Median ±s.d.) | Percentage of female | Age (Mean/Median ±s.d.) | Percentage of person with college degree or above | Independent self-construal | Interdependent self-construal | Influence | Adjustment | Generalized trust |
| --- | --- | --- | --- | --- | --- | --- | --- | --- | --- | --- |
| Beijing | 50 | 19.2/15.0±12.5 | 40.0 | 34.7/33.0±7.6 | 84.0 | 5.2 | 5.2 | 2.5 | 2.1 | 5.1 |
| Shanghai | 100 | 21.3/20.0±12.9 | 37.0 | 33.9/32.5±7.8 | 78.0 | 5.1 | 5.1 | 2.4 | 2.0 | 5.1 |
| Tianjin | 50 | 12.8/10.0±8.6 | 62.0 | 31.5/29.0±8.0 | 68.0 | 4.7 | 5.0 | 2.1 | 2.0 | 4.9 |
| Chongqing | 50 | 12.4/9.5±10.6 | 36.0 | 30.8/31.0±6.6 | 64.0 | 4.7 | 4.8 | 1.8 | 1.7 | 4.7 |
| Anhui | 50 | 11.6/10.0±12.2 | 30.0 | 34.9/34.0±9.4 | 52.0 | 4.8 | 4.7 | 2.0 | 1.9 | 5.1 |
| Fujian | 50 | 11.8/10.0±7.6 | 26.0 | 33.7/33.0±9.4 | 54.0 | 4.8 | 4.8 | 1.8 | 1.6 | 4.9 |
| Gansu | 100 | 15.7/6.0±66.0 | 38.0 | 28.4/26.0±8.3 | 71.0 | 4.7 | 5.1 | 2.0 | 1.9 | 4.9 |
| Guangdong | 50 | 55.4/15.0±240.3 | 28.0 | 33.2/32.0±7.2 | 62.0 | 5.0 | 5.0 | 2.3 | 2.0 | 5.1 |
| Guizhou | 50 | 9.7/7.0±10.1 | 42.0 | 32.8/28.5±12.3 | 56.0 | 4.7 | 4.9 | 1.7 | 1.7 | 4.6 |
| Hainan | 50 | 11.0/6.5±12.5 | 50.0 | 26.8/24.5±6.5 | 66.0 | 4.7 | 4.7 | 1.8 | 1.7 | 5.0 |
| Hebei | 50 | 12.7/10.0±12.4 | 40.0 | 34.7/33.0±7.1 | 68.0 | 4.9 | 5.1 | 2.3 | 2.0 | 5.3 |
| Heilongjiang | 50 | 11.5/8.0±10.4 | 44.0 | 35.6/33.0±8.8 | 64.0 | 4.8 | 4.8 | 2.0 | 1.7 | 5.1 |
| Henan | 50 | 10.7/9.0±10.3 | 24.0 | 34.2/31.0±9.6 | 52.0 | 4.8 | 5.0 | 2.1 | 2.0 | 5.1 |
| Hubei | 50 | 52.8/10.0±281.4 | 48.0 | 32.7/31.5±7.8 | 62.0 | 4.7 | 4.8 | 2.0 | 1.8 | 5.0 |
| Hunan | 50 | 13.5/10.0±10.3 | 44.0 | 32.5/29.5±10.5 | 56.0 | 4.6 | 4.7 | 2.0 | 1.8 | 4.7 |
| Jiangsu | 50 | 12.9/10.0±8.7 | 36.0 | 34.3/34.0±7.6 | 70.0 | 4.8 | 4.9 | 2.1 | 1.8 | 5.1 |
| Jiangxi | 50 | 418.6/9.0±2826.6 | 48.0 | 34.4/31.5±10.8 | 56.0 | 4.9 | 4.9 | 1.9 | 1.8 | 5.0 |
| Jilin | 50 | 9.0/7.0±5.6 | 52.0 | 34.0/34.0±8.9 | 64.0 | 4.8 | 5.0 | 2.0 | 1.8 | 4.9 |
| Liaoning | 50 | 11.1/9.0±6.3 | 38.0 | 37.8/35.5±9.2 | 72.0 | 4.9 | 5.1 | 2.1 | 1.9 | 5.1 |
| Qinghai | 50 | 11.4/10.0±8.1 | 52.0 | 30.9/30.5±6.4 | 66.0 | 5.1 | 5.1 | 2.3 | 2.1 | 5.1 |
| Shaanxi | 50 | 10.3/8.5±7.5 | 26.0 | 32.8/31.0±7.4 | 68.0 | 4.7 | 5.0 | 1.9 | 1.9 | 4.9 |
| Shandong | 50 | 11.3/10.0±6.4 | 20.0 | 36.9/36.0±7.4 | 62.0 | 5.0 | 5.2 | 2.4 | 2.1 | 5.2 |
| Shanxi | 50 | 21.4/6.5±73.0 | 40.0 | 33.6/32.0±8.1 | 48.0 | 4.9 | 4.9 | 1.9 | 1.7 | 4.7 |
| Sichuan | 50 | 13.5/11.5±6.7 | 38.0 | 33.4/32.0±8.4 | 70.0 | 5.0 | 5.0 | 2.3 | 2.0 | 5.3 |
| Yunnan | 50 | 10.1/7.5±7.6 | 52.0 | 31.6/30.0±9.0 | 60.0 | 4.7 | 4.8 | 2.1 | 1.8 | 4.9 |
| Zhejiang | 50 | 16.2/11.5±14.1 | 32.0 | 33.6/32.0±7.0 | 68.0 | 4.8 | 4.9 | 2.2 | 2.0 | 5.1 |
| Guangxi Zhuang | 50 | 11.8/9.0±11.0 | 34.0 | 31.1/30.0±7.0 | 50.0 | 5.0 | 5.0 | 2.2 | 1.9 | 4.8 |
| Inner Mongolia | 50 | 10.3/8.0±7.6 | 40.0 | 33.7/30.5±9.3 | 52.0 | 4.9 | 5.1 | 2.0 | 1.8 | 5.2 |
| Ningxia | 50 | 12.0/9.0±11.6 | 58.0 | 30.8/27.0±10.5 | 74.0 | 4.8 | 5.0 | 2.3 | 2.0 | 5.1 |
| Shinjang Uyghur | 50 | 9.9/7.5±8.4 | 52.0 | 32.3/29.5±8.7 | 72.0 | 4.8 | 4.9 | 1.9 | 1.8 | 5.1 |
| Xizang | 50 | 14.4/13.0±10.0 | 56.0 | 30.9/29.5±6.2 | 68.0 | 5.2 | 5.2 | 2.7 | 2.2 | 5.2 |

# References

Shimizu, H., Murayama, A., & Daibo, I. (2006). Analyzing the interdependence of group communication (1): Application of hierarchical analysis into communication data. *IEICE Technical Report (Human communication science)*, *106*, 1-6.
